# Editorial: Training, performance, and dynamic capabilities: new insights from absorptive, innovative, adaptative, and learning capacities

**DOI:** 10.3389/fpsyg.2023.1176275

**Published:** 2023-06-15

**Authors:** Benito Yáñez-Araque, Juan Moreno-Garcia, Felipe Hernández-Perlines

**Affiliations:** ^1^Department of Business Administration, Faculty of Law and Social Science, University of Castilla-La Mancha, Toledo, Spain; ^2^Department of Information Systems and Technologies, School of Industrial and Aerospace Engineering, University of Castilla-La Mancha, Toledo, Spain

**Keywords:** training, education, performance, learning, innovation, absorptive capacity, adaptative capacity

The coronavirus disease 2019 (COVID-19) pandemic has precipitated the adoption of online teaching-learning methodologies at all educational levels worldwide, affecting all types of training (formal, non-formal, compulsory, non-compulsory, and lifelong). This has given rise to a seemingly new debate on the effectiveness of training that the call for face-to-face training has provoked. However, this debate, apart from the online face-to-face dilemma or their combination, is familiar.

The problem of the transfer of training has been of concern to both academics and practitioners. However, only a small percentage of what is learned during training applies to jobs. Consequently, there is a paradox, an explanatory gap in the relationship between training and performance. With the transfer of training, training efforts can contribute to organizational effectiveness (Kozlowski et al., 2000). However, how, or by what mechanism, does training transfer happen? What is the real relationship?

There is a need for new approaches to training that are different from conventional approaches, which will make it possible to explain and implement the education and training systems that produce a transfer.

In this sense, we found an innovative proposal: a dynamic capability-based view of training (Hernández-Perlines and Yáñez-Araque, 2015; Hernández-Perlines et al., 2016a,b; Yáñez-Araque et al., 2017). Henseler and Schuberth (2020) recently cited this proposal as a novel application of educational management.

There has been exponential growth in the number of publications on dynamic capabilities in the last two decades, and it continues to be one of the most prolific streams (Albort-Morant et al., 2018). Originally, Teece et al. (1997, p. 516) defined dynamic capabilities as the ability of a firm to integrate, build, and reconfigure internal and external competencies to address rapidly changing environments. However, dynamic capabilities theory has some aspects related to its conceptualization that could be clearer, specifically to the factors that compose it.

In the literature, we can find the identification of up to four main components of dynamic capabilities that explain the mechanisms linking the advantages of internal resources to the external market-based competitive advantage of firms: adaptive capacity or flexibility, absorptive capacity, and innovative capacity, which are correlated (Wang and Ahmed, 2007), as well as learning capacity, which was considered by Zollo and Winter (2002). This dynamic capability framework includes absorptive capacity, innovative capacity, flexibility, and learning.

Yáñez-Araque et al. (2017) focused on absorptive and innovative dynamic capacities to explain the transfer process from training to results. It is difficult for training to affect outcomes directly when it is performed through a chain of intermediate variables. Although the study was conducted in a business environment, it can be perfectly extrapolated to the field of education. First, training is indirectly related to performance through absorptive capacity followed by innovative capacity. Training efforts do not translate into tangible benefits if the dynamic absorptive and innovative capabilities are not mediated.

States invest in public education policies to improve the welfare of their citizens. The greatest asset of an organization is its people. Despite evidence of the benefits of training for individuals, teams, organizations, and society (Aguinis and Kraiger, 2009), there is a belief that a student is trained at the individual level with the expectation of a better job and a better future salary. An organization invests in training its workers with expectations to improve its productivity and results. However, more than the mere introduction of training is required to improve performance. The expected benefits of training cannot be obtained without mediating absorptive and innovative capabilities.

Other training problems related to the appropriability of training (e.g., retention of best-trained employees or avoiding brain drain from a country), depreciation of training, technological advances, and issues of equality, diversity, and inclusion are also considered.

The papers selected in this research address the latest research framed in new approaches to training and education from the perspective of public policy, prospective organizations, and individuals. Considering the role of higher education, Yahiaoui et al. investigated the impact of total quality management practices in higher education institutions; Sánchez-Prieto et al. and Procopio et al. focused on novel approaches in higher education and more active and participatory teaching innovation methods. The authors presented a competitive debate regarding gamification among teams with university students, a didactic experience of implementing a methodological approach based on cooperative learning, and a review of the literature on neuroeducation. Gradellini et al. examined the content and knowledge of cultural competence and intercultural communication offered in higher education in nursing courses. Barba-Sánchez et al. developed a model for assessing the impact of secondary school information technology (IT) capacities on smart city business development based on the IMD Smart City Index, PISA, and World Bank reports. Gómez-Cantarino et al. presented a narrative review of health education models, conceptual frameworks, and the importance of information and communication technology (ICT) in a transdisciplinary approach to child abuse. The impact of human resource management practices was discussed by Xie et al., where the impact of human resource management practices and training and development types in multinational enterprises was considered; Xiao et al. studied how supervisors' developmental feedback affects creativity at the team level. Noman et al. examined the moderating role of cross-cultural training in the adjustment of self-initiated and organizational expatriates. Based on dynamic capability theory, Wang et al. researched the effect of a high-performance work system on organizational performance, the mediating role of strategic flexibility, and the moderating role of an enterprise's social network in this relationship. Zhiqiang et al. examined how high-performance work practices (HPWPs) affected employees' in-role performance (EIRP) and task performance (ETP) during the COVID-19 pandemic.

Understanding the state of research on organizations is fundamental to this special issue. Peng et al. conducted a bibliometric study on innovation research in organizations within the three levels (i.e., individual, work team, and organizational) using the CiteSpace software to analyze 6,354 academic articles from the year 2000 to 2020. Respect for ecology within organizations is important in both training and education. Baeshen et al. discussed a study to develop a comprehensive model by integrating the natural resource-based view (NRBV) and triple bottom line (TBL) framework. The influence of absorptive capacity and innovativeness on business performance was analyzed by Sancho-Zamora et al., considering the relationships between the different dimensions of absorptive capacity and innovativeness.

This research combines high-quality research with different quantitative and qualitative methodologies and mixed-methods studies. It also includes reviews that follow quality criteria, and research projects that adopt multisectoral and transdisciplinary approaches.

## Author contributions

BY-A, JM-G, and FH-P contributed to conception and design of the study and wrote sections of the manuscript. BY-A wrote the first draft of the manuscript. All authors contributed to manuscript revision, read, and approved the submitted version.
